# Marked Hypergastrinemia and Extreme Erythrocyte Sedimentation Rate Elevation in Autoimmune Atrophic Gastritis with Severe Iron Deficiency Anemia: A Case Report

**DOI:** 10.1002/ccr3.73865

**Published:** 2026-11-03

**Authors:** Hatem Mousa Taha, Khaled Hatem Taha

**Affiliations:** ^1^ Faculty of Medicine and Health Sciences An‐Najah National University Nablus Palestine; ^2^ Department of Internal Medicine Palestine Medical Complex Ramallah Palestine

**Keywords:** autoimmune atrophic gastritis, case report, erythrocyte sedimentation rate, hypergastrinemia, iron deficiency anemia

## Abstract

Autoimmune atrophic gastritis causes loss of oxyntic glands, reduced acid secretion, compensatory hypergastrinemia, and impaired iron and vitamin B12 absorption. A 46‐year‐old woman with minimal gastrointestinal symptoms had fasting serum gastrin > 1800 ng/L, an erythrocyte sedimentation rate of 109 mm/h, hemoglobin 8.2 g/dL, and microcytosis. Anti‐parietal cell and intrinsic factor antibodies were positive. Endoscopy and Sydney‐protocol biopsies showed oxyntic‐restricted atrophy, preserved antral mucosa, and linear‐to‐micronodular enterochromaffin‐like‐cell hyperplasia; C‐reactive protein was normal, and computed tomography showed no pancreatic or duodenal mass. After iron and vitamin B12 replacement, hemoglobin rose to 11.0 g/dL, and the erythrocyte sedimentation rate fell to 60 mm/h, whereas gastrin remained > 1800 ng/L at the measured 12‐week follow‐up. The findings support autoimmune atrophic gastritis as the explanation for the hypergastrinemia and an anemia‐associated contribution to the raised erythrocyte sedimentation rate. This case reinforces the need to interpret severe hypergastrinemia in relation to gastric acid status and extreme erythrocyte sedimentation rate values in relation to hematological factors.

## Key Clinical Message

In autoimmune atrophic gastritis, marked hypergastrinemia and an extreme erythrocyte sedimentation rate may coexist when severe iron deficiency anemia is present. Integrating gastric acid status, autoimmune serology, histology, C‐reactive protein, and serial blood counts may reduce misclassification as gastrinoma or systemic inflammation.

## 1 Introduction

Autoimmune atrophic gastritis (AAG) is a chronic immune‐mediated disorder that targets the oxyntic mucosa, causing parietal‐cell loss, reduced acid secretion, compensatory hypergastrinemia, and impaired iron and vitamin B12 absorption [1, 2, 3, 4, 5, 6]. Marked hypergastrinemia may prompt evaluation for gastrinoma and Zollinger‐Ellison syndrome (ZES), whereas an erythrocyte sedimentation rate (ESR) greater than 100 mm/h commonly raises concern for infection, malignancy, or systemic inflammatory disease [7, 8, 9, 10].

Severe hypergastrinemia and iron deficiency anemia are established manifestations of AAG [1, 2, 3, 4, 5, 6, 11]. The educational value of this report is therefore not a unique association, but the interpretive challenge created when these recognized consequences coexist with an extreme ESR and a clinically mild presentation. We describe the diagnostic reasoning and serial response to treatment while acknowledging the absence of direct gastric pH measurement and complete functional testing for ZES.

## 2 Case History and Examination

### 2.1 Clinical Presentation

A 46‐year‐old woman was referred for evaluation of unexplained laboratory abnormalities. She reported longstanding fatigue and recurrent iron deficiency anemia managed intermittently with oral iron supplements. There was no history of peptic ulcer disease, inflammatory bowel disease, renal disease, or malignancy. Symptoms were limited to occasional mild dyspepsia. She denied abdominal pain, secretory diarrhea, weight loss, or gastrointestinal bleeding. No features attributable to gastric acid hypersecretion were present, which reduced the clinical probability of ZES. Interview and pharmacy records documented no current or prior proton pump inhibitor (PPI) or H2‐receptor antagonist use.

### 2.2 Physical Examination

Vital signs: blood pressure 118/74 mmHg, heart rate 96 beats per minute, temperature 36.8°C, oxygen saturation 98% on room air. Examination revealed pallor of the conjunctivae and nail beds consistent with anemia. There was no lymphadenopathy, hepatosplenomegaly, or abdominal tenderness. Neurological examination was unremarkable. The overall presentation of a systemically well patient with signs of chronic anemia alone was notably at odds with the severity of the laboratory abnormalities.

### 2.3 Initial Laboratory Evaluation

Laboratory investigations revealed hemoglobin 8.2 g/dL, mean corpuscular volume (MCV) 68 fL, ESR 109 mm/h by the Westergren method (reference for women aged 40 to 50 years, 20 mm/h or less), and fasting serum gastrin > 1800 ng/L (reference < 100 ng/L). Gastrin was measured after a minimum 12 h overnight fast; interview and pharmacy records documented no PPI or H2‐receptor antagonist use for at least 4 weeks before sampling. The electrochemiluminescence immunoassay was performed on the Roche Diagnostics Cobas platform, with an intra‐assay coefficient of variation < 5%. A repeat measurement was 1849 ng/L. Gastrin values are reported in ng/L, equivalent to pg/mL. Severe hypergastrinemia and an extreme ESR in a patient with microcytic anemia and minimal symptoms warranted integrated evaluation.

## 3 Differential Diagnosis, Investigations, and Treatment

### 3.1 Differential Diagnosis

A structured differential diagnosis was pursued for both the severe hypergastrinemia and the extreme ESR elevation. The differential diagnoses for severe hypergastrinemia and extreme ESR elevation are summarized in Tables 1 and 2, respectively.

**TABLE 1:** Differential diagnosis of severe hypergastrinemia (serum gastrin > 1000 ng/L).

Condition	Key clue	Why considered	Finding in this case
Zollinger–Ellison syndrome	Marked fasting hypergastrinemia with gastric acid hypersecretion; ulcer disease or secretory diarrhea	Fasting gastrin > 1800 ng/L	Considered unlikely: no acid‐hypersecretory phenotype and CT negative; gastric pH and secretin testing unavailable
Autoimmune atrophic gastritis	Oxyntic atrophy, reduced acid secretion, APCA/IFA positivity	Marked gastrin with iron and vitamin B12 deficiency	Supported: positive antibodies, corpus/fundus atrophy, preserved antrum, and ECL‐cell hyperplasia
PPI‐associated hypergastrinemia	Current or recent acid‐suppressive therapy	Common acquired cause of hypergastrinemia	Not supported: no PPI or H2‐receptor antagonist exposure by interview and pharmacy records
*H. pylori* ‐associated atrophic gastritis	Evidence of *H. pylori* with multifocal atrophy	Atrophic‐gastritis differential	Not supported: rapid urease testing and histology were negative
Chronic kidney disease	Reduced renal clearance with impaired renal function	Hypergastrinemia differential	Not supported: creatinine and estimated glomerular filtration rate were normal

Abbreviations: AAG, autoimmune atrophic gastritis; APCA, anti‐parietal cell antibodies; CT, computed tomography; IFA, intrinsic factor antibodies; PPI, proton pump inhibitor; ZES, Zollinger‐Ellison syndrome.

**TABLE 2:** Differential diagnosis of extreme erythrocyte sedimentation rate (ESR) elevation (greater than 100 mm/h).

Condition	Key clue	Why considered	Finding in this case
Plasma‐cell dyscrasia	Monoclonal protein, bone pain, renal dysfunction, or hypercalcaemia	Extreme ESR with anemia	No monoclonal band on SPEP and no related clinical features
Active infection or inflammation	Systemic illness with an acute‐phase response	Common cause of extreme ESR	No clinical evidence at assessment: afebrile, clinically stable, and CRP normal
Systemic autoimmune disease	Compatible symptoms, examination, and serology	Common cause of extreme ESR	No supporting clinical or laboratory features; CRP normal
Severe iron deficiency anemia	Microcytosis, low ferritin, and possible ESR/CRP discordance	Hemoglobin 8.2 g/dL and ferritin 8 micrograms/L	Supported as a major contributor: ESR declined during hematological recovery while CRP remained normal
Malignancy	Constitutional features, lymphadenopathy, or imaging abnormality	Extreme ESR differential	No supporting CT or clinical findings
Liver or kidney disease	Abnormal organ function or altered plasma proteins	Can influence ESR and gastrin interpretation	Not supported: hepatic and renal function were normal

Abbreviations: CRP, C‐reactive protein; CT, computed tomography; ESR, erythrocyte sedimentation rate; SPEP, serum protein electrophoresis.

For the hypergastrinemia, the absence of peptic ulcer disease, secretory diarrhea, reflux complications, hypertrophic gastric folds, or oesophagitis reduced the clinical probability of ZES. Iron deficiency anemia and borderline vitamin B12 deficiency suggested a low‐acid gastric mechanism, although gastric pH was not measured. PPI‐associated hypergastrinemia was not supported because no acid‐suppressive medication exposure was documented. *H. pylori* ‐associated atrophic gastritis was unlikely because rapid urease testing and histology were negative (Section 3.3). Reduced renal gastrin clearance was not supported because renal function was normal.

For the ESR elevation: multiple myeloma, severe infection, systemic autoimmune disease, severe anemia, and chronic kidney disease were all systematically evaluated.

### 3.2 Laboratory and Immunological Investigations

Targeted investigations demonstrated: vitamin B12 137 pmol/L (borderline low); APCA: positive; IFA: positive; serum ferritin 8 μg/L; serum iron 3.9 μmol/L; transferrin saturation 7%; CRP within normal limits (less than 5 mg/L); serum protein electrophoresis (SPEP) with no monoclonal band; and normal renal and hepatic function.

The discordance between a normal C‐reactive protein (CRP) and an extreme ESR suggested that non‐inflammatory influences on ESR, including severe anemia, should be considered. Because CRP and ESR respond to different determinants, neither result alone excludes infection, malignancy, or autoimmune disease. The absence of a monoclonal band on serum protein electrophoresis and the unremarkable clinical assessment further reduced concern for plasma‐cell dyscrasia or active systemic inflammation [9, 10].

### 3.3 Endoscopic and Histopathological Findings

Upper endoscopy showed macroscopically atrophic mucosa in the gastric corpus and fundus with preservation of the antral mucosa. Systematic Sydney‐protocol biopsies showed mucosal atrophy confined to the oxyntic mucosa, with near‐complete loss of oxyntic glands; histologically normal antral mucosa with intact G cells and no significant inflammation or atrophy; chronic lamina propria inflammation confined to the oxyntic mucosa; and linear‐to‐micronodular enterochromaffin‐like (ECL)‐cell hyperplasia confirmed by chromogranin A immunostaining [12, 13]. No dysplasia or neuroendocrine tumor was identified.

*Helicobacter pylori* was assessed by rapid urease testing of antral and corpus biopsies and by histological examination of Giemsa‐stained sections from both sites; neither method detected the organism, making active *H. pylori* infection unlikely. Positive APCA and IFA together with oxyntic‐restricted atrophic gastritis and normal antral mucosa supported the diagnosis of AAG [1, 2].

### 3.4 Imaging and Functional Testing

Contrast‐enhanced abdominal computed tomography (CT) showed no pancreatic or duodenal mass, lymphadenopathy, or visceral lesion. Direct gastric pH measurement, secretin stimulation testing, endoscopic ultrasonography (EUS), and somatostatin‐receptor imaging were not locally available. ZES therefore could not be excluded through the complete conventional functional pathway. Nevertheless, the histological diagnosis of AAG, positive gastric autoantibodies, absence of acid‐hypersecretory features, and negative CT made AAG the better‐supported explanation for the hypergastrinemia [7, 8, 14, 15].

### 3.5 Treatment

Treatment was started for AAG with iron deficiency anemia and borderline vitamin B12 deficiency. Oral ferrous sulphate 200 mg three times daily (65 mg elemental iron per dose) was prescribed for 12 weeks. Intramuscular cyanocobalamin 1000 micrograms was given weekly for 4 weeks and then monthly; positive intrinsic factor antibodies supported autoimmune loss of intrinsic factor. A 12‐month follow‐up endoscopy was planned after clinical review because of the extent of atrophy and ECL‐cell hyperplasia [3, 12, 13, 16].

## 4 Outcome and Follow‐Up

At Week 6, hemoglobin was 9.8 g/dL, mean corpuscular volume (MCV) 72 fL, ESR 82 mm/h, and ferritin 12 micrograms/L; CRP remained normal, and gastrin was not measured. At Week 12, hemoglobin had improved from 8.2 to 11.0 g/dL, MCV from 68 to 76 fL, ESR from 109 to 60 mm/h, ferritin from 8 to 18 micrograms/L, and vitamin B12 from 137 to 320 pmol/L. Fasting serum gastrin remained > 1800 ng/L at the measured Week 12 follow‐up.

The parallel improvement in hemoglobin and ESR during iron replacement, with CRP normal throughout, supported an important anemia‐associated contribution to the raised ESR. The persistently high gastrin at the measured follow‐up remained consistent with sustained loss of acid‐mediated feedback, although gastric acid output was not measured directly.

The changes in laboratory parameters over time are shown in Figure 1, and the baseline and follow‐up values are summarized in Table 3.

**FIGURE 1:**
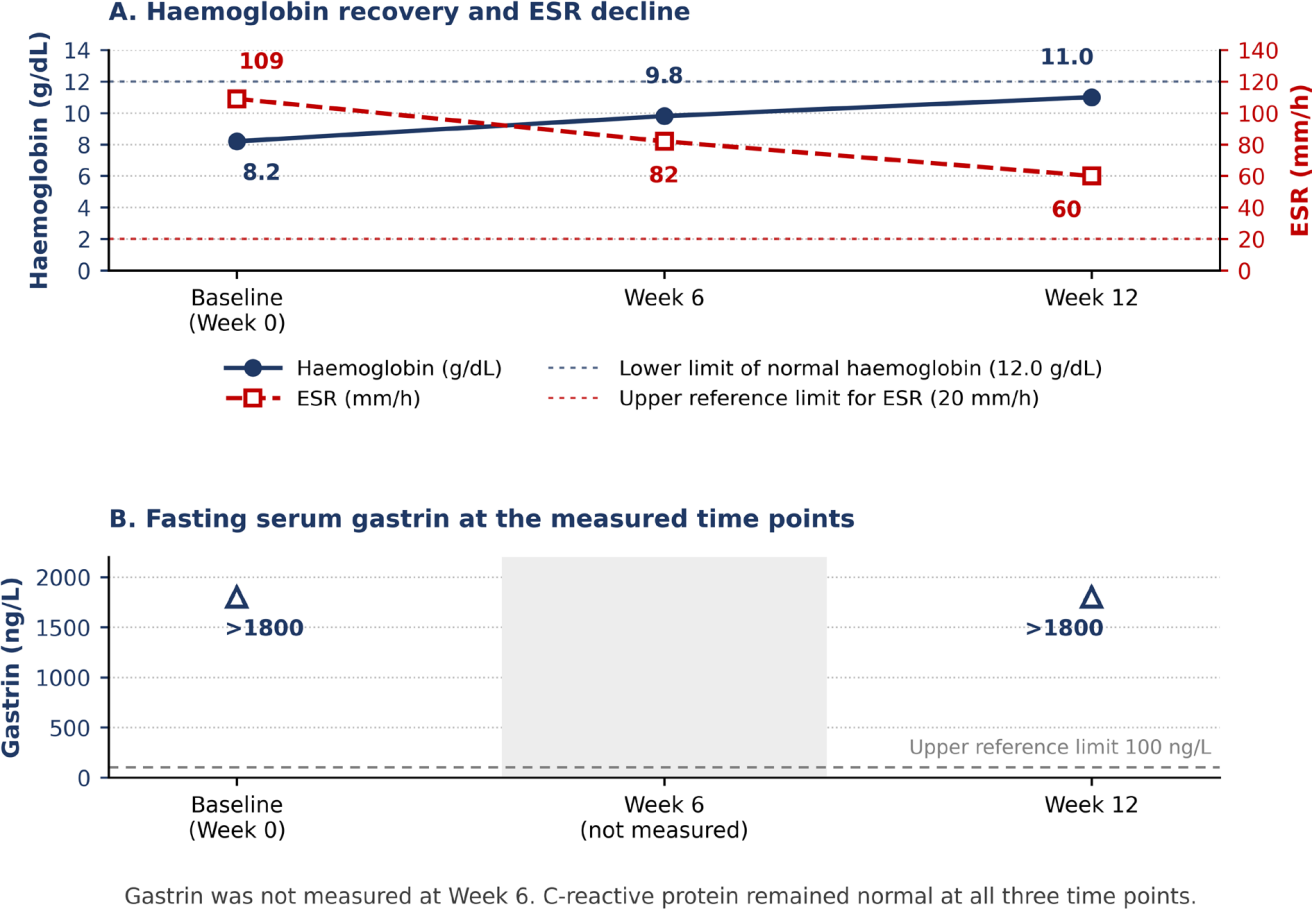
Serial laboratory values during oral iron supplementation. Panel A shows hemoglobin increasing from 8.2 g/dL at baseline to 9.8 g/dL at Week 6 and 11.0 g/dL at Week 12, while erythrocyte sedimentation rate (ESR) declined from 109 to 82 and 60 mm/h, respectively. Panel B shows fasting serum gastrin > 1800 ng/L at baseline and Week 12. Gastrin was not measured at Week 6 and is displayed as missing. C‐reactive protein remained normal throughout. The parallel improvement in hemoglobin and ESR supports an anemia‐associated contribution to ESR elevation but does not establish causality. CRP, C‐reactive protein; ESR, erythrocyte sedimentation rate.

**TABLE 3:** Baseline and follow‐up laboratory parameters.

Parameter	Reference range	Baseline (Week 0)	Week 6^a^	Week 12	Clinical interpretation
Hemoglobin (g/dL)	12.0–16.0	8.2 ↓	9.8 ↑	11.0 ↑	Progressive improvement with oral iron supplementation
Mean corpuscular volume, MCV (fL)	80–100	68 ↓	72 ↑	76 ↑	Microcytosis improving progressively
Erythrocyte sedimentation rate, ESR (mm/h, Westergren method)^b^	≤ 20	109 ↑	82 ↓	60 ↓ (−45%)	Progressive decline during hematological recovery; supports an anemia‐associated contribution but does not establish sole causation
Serum gastrin^c^ (ng/L, fasting)	< 100	> 1800 ↑	Not measured	> 1800 (persistently elevated)	Persistently > 1800 ng/L at measured baseline and Week 12 time points; consistent with sustained loss of acid feedback
C‐reactive protein, CRP (mg/L)	< 5	Normal (< 5)	Normal (< 5)	Normal (< 5)	Normal CRP throughout made a strong systemic acute‐phase response less likely but did not exclude inflammatory disease
Serum ferritin (μg/L)	12–150	8 ↓	12 ↑	18 ↑	Progressive iron store replenishment
Serum iron (μmol/L)	10.7–32.2	3.9 ↓	Not repeated	Not repeated	Severe iron deficiency at presentation
Transferrin saturation (%)	20–45	7 ↓	Not repeated	Not repeated	Supports iron deficiency anemia
Vitamin B12 (pmol/L)	148–616	137 ↓ (borderline)	Not repeated	320 ↑	Normalized following intramuscular cyanocobalamin replacement
APCA serology^d^	Negative	Positive	—	—	Supports autoimmune gastric disease
IFA serology^d^	Negative	Positive	—	—	Supports autoimmune loss of intrinsic factor and risk of vitamin B12 deficiency
Serum protein electrophoresis (SPEP)	No M‐protein	No monoclonal band	—	—	No monoclonal band detected; reduced support for plasma‐cell dyscrasia

Abbreviations: CRP, C‐reactive protein; ECLIA, electrochemiluminescence immunoassay; ESR, erythrocyte sedimentation rate; MCV, mean corpuscular volume; PPI, proton pump inhibitor; SPEP, serum protein electrophoresis.

^a^ Week 6 values were obtained from the routine follow‐up clinical records. Hemoglobin and ESR were measured at a standard review visit.

^b^ ESR reference ranges by age and sex (Westergren method): women 20–50 years, ≤ 20 mm/h; women > 50 years, ≤ 30 mm/h; men 20–50 years, ≤ 15 mm/h; men > 50 years, ≤ 20 mm/h. This patient (46‐year‐old woman) had a reference upper limit of 20 mm/h; her presenting ESR represented a 5.5‐fold elevation above the age‐ and sex‐specific upper reference limit.

^c^ Fasting serum gastrin measured after a minimum 12 h overnight fast with no proton pump inhibitor (PPI) or H2‐receptor antagonist use for at least 4 weeks prior to sampling. Assay: electrochemiluminescence immunoassay (ECLIA), Roche Diagnostics Cobas platform. All values reported in ng/L, which is equivalent to pg/mL (1 ng/L = 1 pg/mL). Confirmatory repeat measurement: 1849 ng/L.

^d^ APCA, anti‐parietal cell antibodies; IFA, intrinsic factor antibodies.

The patient was counseled regarding the diagnosis of autoimmune atrophic gastritis, the need for lifelong vitamin B12 replacement, and the importance of periodic endoscopic surveillance.

## 5 Discussion

### 5.1 Interpreting Severe Hypergastrinemia in Autoimmune Atrophic Gastritis

Hypergastrinemia, iron deficiency, vitamin B12 deficiency, and few or nonspecific upper gastrointestinal symptoms are recognized features of AAG [1, 2, 3, 4, 5, 6, 11, 17, 18, 19, 20, 21]. The present case does not establish a new association. Instead, it shows how established manifestations can converge to resemble a gastrinoma or systemic inflammatory disorder when laboratory values are considered without the clinical and histological context.

The mechanism of hypergastrinemia differs fundamentally between ZES and AAG. In ZES, inappropriate gastrin secretion occurs in the setting of gastric acid hypersecretion. In AAG, parietal‐cell loss reduces acid‐mediated inhibition of antral G cells, so gastrin rises as a compensatory response. Gastrin concentration alone cannot distinguish these states; medication exposure, gastric acid status, serology, endoscopy, and histology must be integrated [3, 7, 8, 14, 15].

In this patient, the absence of ulcer disease, secretory diarrhea, reflux complications, or endoscopic features of acid hypersecretion reduced the clinical probability of ZES. Positive anti‐parietal cell and intrinsic factor antibodies together with oxyntic‐restricted atrophy and preserved antral mucosa provided direct support for AAG [1, 2, 3].

Chronic hypergastrinemic stimulation is also associated with linear and micronodular ECL‐cell hyperplasia and an increased risk of Type 1 gastric neuroendocrine neoplasia, supporting endoscopic follow‐up [3, 12, 13]. Figures S1 and S2 summarize the diagnostic reasoning and pathophysiology as illustrative teaching tools; they are not validated or evidence‐graded diagnostic algorithms.

### 5.2 Extreme ESR Elevation in Iron Deficiency Anemia

The ESR is influenced by plasma proteins and erythrocyte characteristics [9, 10]. Anemia can increase sedimentation by reducing hematocrit and altering the physical conditions that govern erythrocyte aggregation. Gastric hypoacidity also impairs non‐haem iron absorption, making iron deficiency a well‐recognized early hematological manifestation of AAG [4, 5, 6, 21].

In this case, a normal CRP and absence of systemic illness made a strong acute‐phase response less likely but did not independently exclude inflammatory disease. The stepwise fall in ESR as hemoglobin and ferritin improved strengthened the interpretation that severe iron deficiency anemia materially contributed to the ESR elevation; the observational trajectory does not prove that anemia was the sole cause.

### 5.3 Clinical Integration and Differential Diagnosis

The combined findings made an atrophic, low‐acid mechanism more likely than ZES: the patient had characteristic oxyntic‐restricted atrophy, positive gastric autoantibodies, preserved antral mucosa, and no acid‐hypersecretory phenotype. Similarly, normal CRP, clinical stability, unremarkable organ function, negative CT, and no monoclonal band reduced support for active systemic inflammation, malignancy, or plasma‐cell dyscrasia without absolutely excluding those diagnoses. The clinical lesson is the value of integrating physiology, histology, serology, and longitudinal data rather than interpreting either laboratory value in isolation.

### 5.4 Limitations and Clinical Implications

Direct gastric pH measurement, secretin stimulation testing, EUS, and somatostatin‐receptor imaging were unavailable; therefore, a small gastrinoma was not excluded by the complete functional and imaging pathway, and CT alone cannot exclude a small lesion. The Week 6 data were obtained retrospectively from routine records, and gastrin was not measured at that visit. The observational nature of a single case precludes definitive attribution of the ESR elevation solely to iron deficiency anemia, and the iron deficiency may have been multifactorial because a gynecological contribution was not fully excluded. These limitations support a cautious interpretation: the trajectory is consistent with severe anemia as a major contributor to ESR elevation, while the serological and histological findings establish AAG as the gastric diagnosis.

## 6 Conclusion

Severe hypergastrinemia and iron deficiency anemia are established manifestations of AAG. In this patient, their coexistence with an extreme ESR and normal CRP created a misleading laboratory profile; autoimmune serology, oxyntic‐restricted atrophy, ECL‐cell hyperplasia, and the serial response to replacement therapy supported AAG with an anemia‐associated contribution to ESR elevation. Marked gastrin and ESR values should be interpreted within their acid‐secretory, histological, serological, and hematological context.

## 7 Learning Points

- Marked hypergastrinemia is not specific for gastrinoma. Achlorhydric autoimmune atrophic gastritis is a recognized cause and should be assessed with medication history, gastric acid status where available, serology, endoscopy, and histology.
- An extreme ESR with normal C‐reactive protein warrants assessment of non‐inflammatory determinants, including anemia. Serial hemoglobin, ferritin, CRP, and ESR values may help clarify the contribution of iron deficiency.
- Linear or micronodular ECL‐cell hyperplasia reflects chronic hypergastrinemic stimulation and supports appropriate endoscopic follow‐up for gastric neuroendocrine neoplasia risk.

## Author Contributions

**Hatem Mousa Taha:** conceptualization, investigation, writing – original draft, supervision. **Khaled Hatem Taha:** investigation, data curation, writing – review and editing.

## Funding

The authors have nothing to report.

## Ethics Statement

Written informed consent was obtained from the patient for publication of this case report and accompanying images. Ethics committee review was not required in accordance with local institutional policy. The report was prepared in accordance with the principles of the Declaration of Helsinki and with reference to the CARE reporting guideline.

## Consent

Written informed consent for publication was obtained from the patient.

## Conflicts of Interest

The authors declare no conflicts of interest.

## Supporting information


**Figure S1:** Illustrative teaching framework for evaluating severe hypergastrinemia (serum gastrin > 1000 ng/L). The framework emphasizes medication review and assessment of gastric acid secretory status. Acid hypersecretion supports evaluation for Zollinger–Ellison syndrome, whereas hypo‐ or achlorhydria supports evaluation for autoimmune or other atrophic gastritis using serology and Sydney‐protocol biopsies. When direct acid measurement is unavailable, clinical, serological, endoscopic, and histological findings must be integrated. This author‐created schematic is an illustrative teaching aid, not a validated or evidence‐graded diagnostic algorithm and not a substitute for current guidance. AAG, autoimmune atrophic gastritis; APCA, anti‐parietal cell antibodies; CT, computed tomography; EUS, endoscopic ultrasonography; IFA, intrinsic factor antibodies; MRI, magnetic resonance imaging; PPI, proton pump inhibitor; ZES, Zollinger‐Ellison syndrome.

**Figure S2:** Illustrative teaching schematic of recognized pathways in autoimmune atrophic gastritis. Parietal‐cell loss reduces acid and intrinsic factor, contributing to compensatory hypergastrinemia, ECL‐cell hyperplasia, and impaired absorption of iron and vitamin B12. Severe anemia can influence ESR through hematocrit and erythrocyte aggregation. This author‐created figure is intended for teaching; it is not an evidence‐graded diagnostic algorithm or a quantitative causal model. APCA, anti‐parietal cell antibodies; ECL, enterochromaffin‐like; ESR, erythrocyte sedimentation rate; IFA, intrinsic factor antibodies.

## Data Availability

Data sharing not applicable to this article as no datasets were generated or analyzed during the current study.
