# Supplementary material for: Marked Hypergastrinemia and Extreme Erythrocyte Sedimentation Rate Elevation in Autoimmune Atrophic Gastritis with Severe Iron Deficiency Anemia: A Case Report
Source: Clin Case Rep. 2026 Nov 3;14(11):e73865. doi: 10.1002/ccr3.73865 (PMC13729736; doi:10.1002/ccr3.73865)
Supplement: Supplementary file 1 [file CCR3-14-e73865-s001.pdf]

Supplementary Figure 1.

Illustrative Teaching Framework for Marked Hypergastrinemia (Serum Gastrin >1000 ng/L)

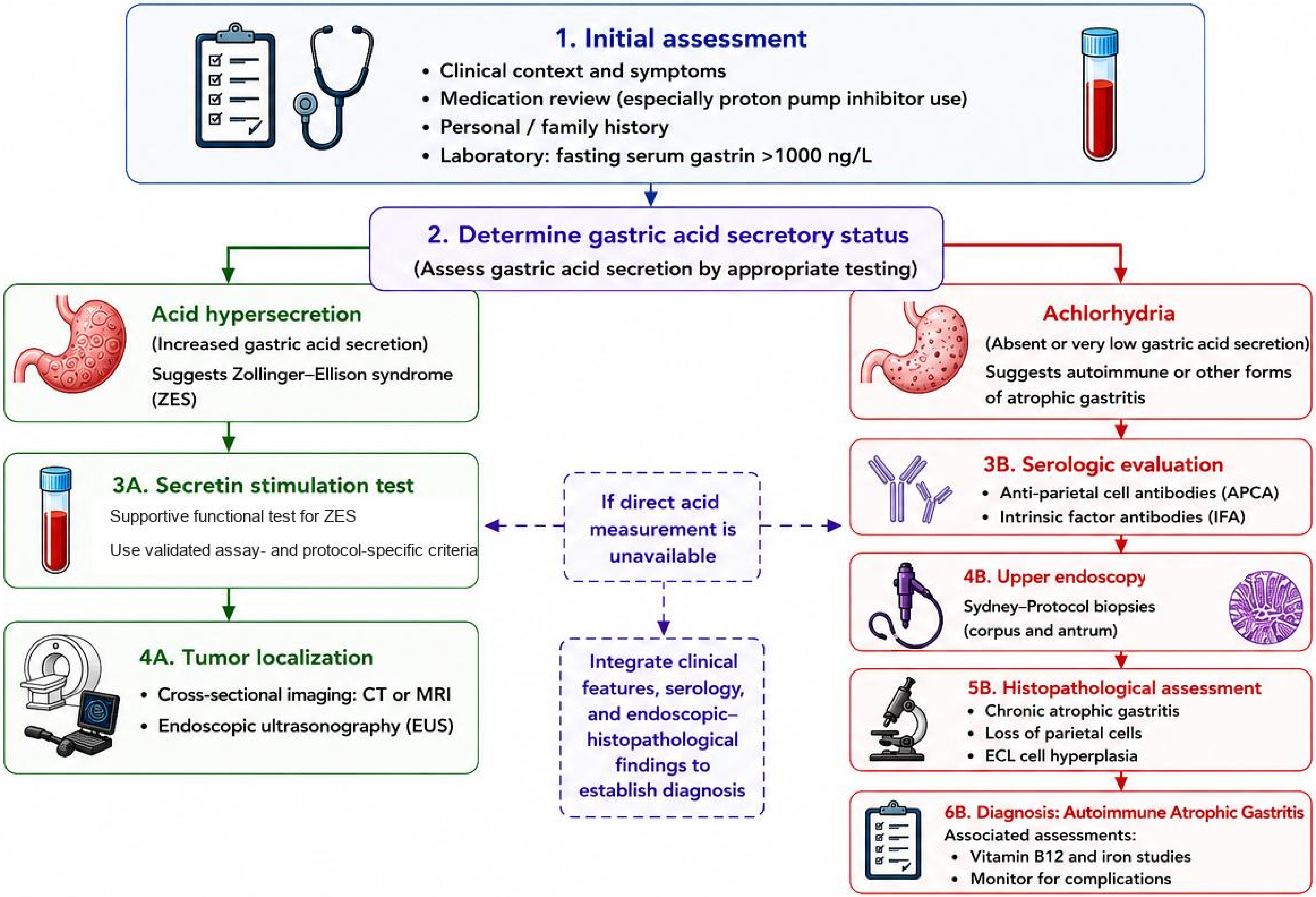

Abbreviations: AAG, autoimmune atrophic gastritis; APCA, anti-parietal cell antibodies; EUS, endoscopic ultrasonography; IFA, intrinsic factor antibodies; PPI, proton pump inhibitor; ZES, Zollinger–Ellison syndrome.

This figure was created and reviewed by the authors for scientific accuracy.

Illustrative teaching tool prepared by the authors; it is not a validated or evidence-graded diagnostic decision rule and does not replace current guidance.
