# Supplementary material for: Marked Hypergastrinemia and Extreme Erythrocyte Sedimentation Rate Elevation in Autoimmune Atrophic Gastritis with Severe Iron Deficiency Anemia: A Case Report
Source: Clin Case Rep. 2026 Nov 3;14(11):e73865. doi: 10.1002/ccr3.73865 (PMC13729736; doi:10.1002/ccr3.73865)
Supplement: Supplementary file 2 [file CCR3-14-e73865-s002.pdf]

Supplementary Figure 2. Parallel Pathophysiological Pathways in Autoimmune Atrophic Gastritis

Recognised consequences are shown as parallel branches, not as a single linear cascade

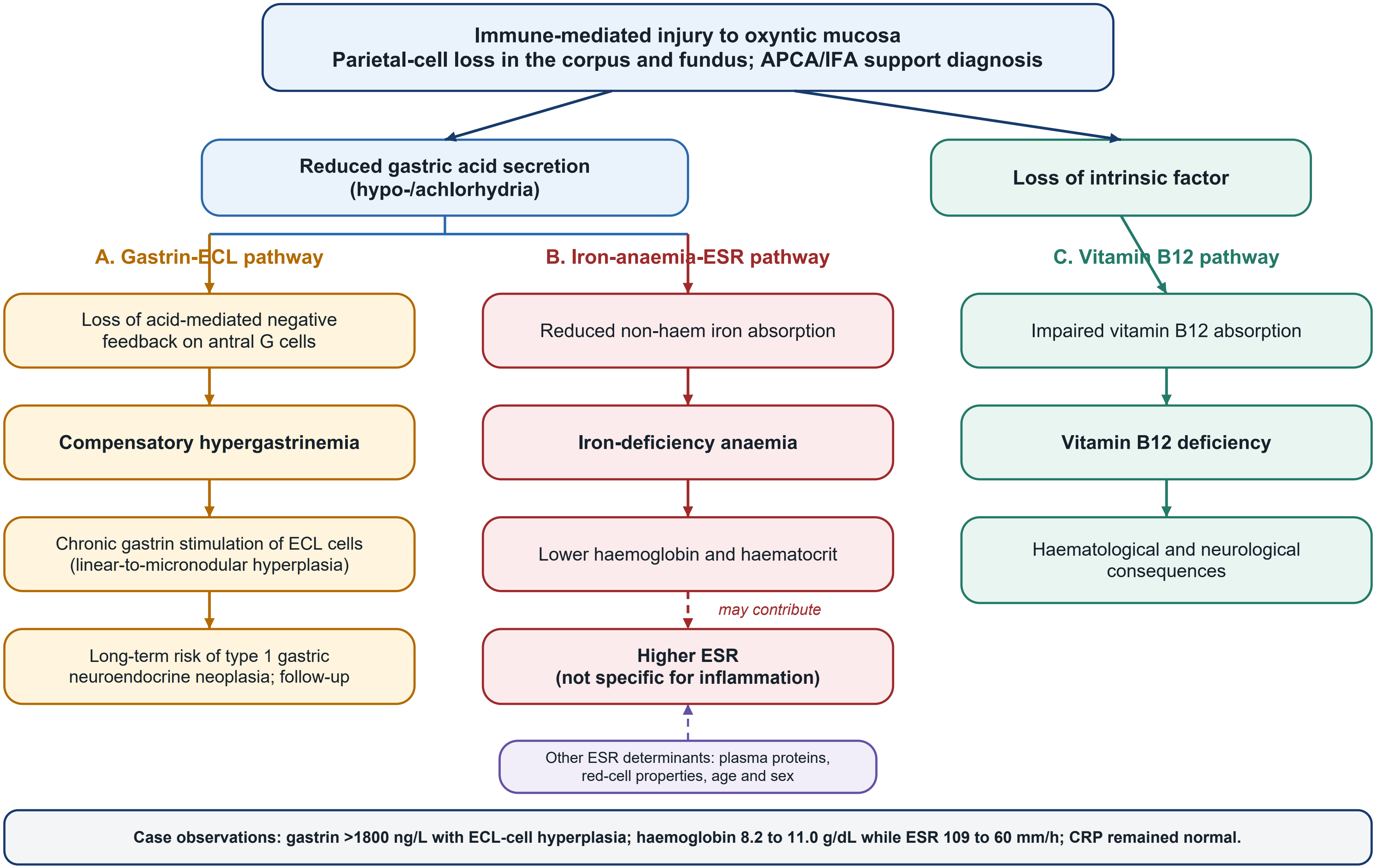

Interpretation: the parallel haemoglobin recovery and ESR decline support an anaemia-associated contribution, but do not establish causality.

Illustrative teaching schematic; not a validated or evidence-graded diagnostic algorithm or a quantitative causal model. Abbreviations: APCA, anti-parietal cell antibodies; CRP, C-reactive protein; ECL, enterochromaffin-like; ESR, erythrocyte sedimentation rate; IFA, intrinsic factor antibodies.
